# The prediction of sagittal chin point relapse following two-jaw surgery using machine learning

**DOI:** 10.1038/s41598-023-44207-2

**Published:** 2023-10-09

**Authors:** Young Ho Kim, Inhwan Kim, Yoon-Ji Kim, Minji Kim, Jin-Hyoung Cho, Mihee Hong, Kyung-Hwa Kang, Sung-Hoon Lim, Su-Jung Kim, Namkug Kim, Jeong Won Shin, Sang-Jin Sung, Seung-Hak Baek, Hwa Sung Chae

**Affiliations:** 1 Department of Orthodontics, Institute of Oral Health Science, Ajou University School of Medicine, Suwon, South Korea; 2 Department of Convergence Medicine, Asan Medical Center, Asan Medical Institute of Convergence Science and Technology, University of Ulsan College of Medicine, Seoul, Korea; 3 Department of Orthodontics, Asan Medical Center, University of Ulsan College of Medicine, Seoul, Korea; 4 Department of Orthodontics, College of Medicine, Ewha Woman’s University, Seoul, Korea; 5 Department of Orthodontics, Chonnam National University School of Dentistry, Gwangju, Korea; 6 Department of Orthodontics, School of Dentistry, Kyungpook National University, Daegu, Korea; 7 Department of Orthodontics, School of Dentistry, Wonkwang University, Iksan, Korea; 8 Department of Orthodontics, College of Dentistry, Chosun University, Gwangju, Korea; 9 Department of Orthodontics, Kyung Hee University School of Dentistry, Seoul, Korea; 10 Department of Orthodontics, School of Dentistry, Dental Research Institute, Seoul National University, Seoul, South Korea; 11 Department of Orthodontics, Gwangmyeong Hospital, Chungang University, Gwangmyeong, Korea

**Keywords:** Biophysics, Computational biology and bioinformatics

## Abstract

The study aimed to identify critical factors associated with the surgical stability of pogonion (Pog) by applying machine learning (ML) to predict relapse following two-jaw orthognathic surgery (2 J-OGJ). The sample set comprised 227 patients (110 males and 117 females, 207 training and 20 test sets). Using lateral cephalograms taken at the initial evaluation (T0), pretreatment (T1), after (T2) 2 J-OGS, and post treatment (T3), 55 linear and angular skeletal and dental surgical movements (T2-T1) were measured. Six ML modes were utilized, including classification and regression trees (CART), conditional inference tree (CTREE), and random forest (RF). The training samples were classified into three groups; highly significant (HS) (≥ 4), significant (S) (≥ 2 and < 4), and insignificant (N), depending on Pog relapse. RF indicated that the most important variable that affected relapse rank prediction was ramus inclination (RI), CTREE and CART revealed that a clockwise rotation of more than 3.7 and 1.8 degrees of RI was a risk factor for HS and S groups, respectively. RF, CTREE, and CART were practical tools for predicting surgical stability. More than 1.8 degrees of CW rotation of the ramus during surgery would lead to significant Pog relapse.

## Introduction

Orthognathic surgery is performed to overcome skeletal discrepancies, obtain esthetics, and achieve normal occlusion. However, unstable outcomes often require dental compensation during postoperative orthodontic treatment and other surgical procedures^1^. Surgical instability, including hierarchy in post-surgical stability, is well established based on the surgical direction. Changes > 2 mm or 2° were defined as moderately unstable, and 4 > mm or 4° were highly unstable^2–4^. A comprehensive report on hierarchy^5^ indicated that post-surgical instability after mandibular setback was related to "A technical problem," which meant that the chin occasionally underwent clockwise (CW) rotation during the operation, and later the pterygomassetreic sling induced the opposite direction even with rigid fixation. The quantity of CW rotation of the proximal segment was correlated with the linear measurement of pogonion (Pog)^6^. Although two-jaw orthognathic surgery(2 J-OGS) was expected to overcome this situation, the proximal segment counter CW rotation after surgery, measured as ramus inclination (RI), was significantly associated with the amount of mandibular relapse^7^. Based on the literature above, the major relapse occurred during CW rotation of the ramus (proximal segment) during surgery, which was related to the forward movement of the Pog after surgery. Therefore, training a dataset by including pre-operative (T1) and post-operative RI change (T2, and six to eight weeks later) to a machine learning (ML) algorithm may lead to predicting the change in Pog during retention in the testing set.

Artificial intelligence (AI) refers to the development of computer systems that can perform tasks that require human intelligence. ML is a subfield of AI that focuses on devising algorithms and statistical models that computers can use to "learn" from data without explicit programming. Deep learning is a subset of ML that uses artificial neural networks inspired by the structure and function of the human brain to process and analyze large amounts of data^8^. Studies on ML and deep learning in the field of temporomandibular joint (TMJ) in the dental orthodontic department have been reported^9–13^. Jung stated that it is possible to classify extraction versus non-extraction with a 93% success rate using ML^9^. Etemd reported the ranking factors determining the extraction using random forest (RF)^10^. Li suggested that the K-Nearest Neighbors (KNN) method was the best model for distinguishing between extraction and non-extraction, extraction patterns, and anchorage determination^11^. Fang used multivariate logistic regression to detect cephalometric variables associated with degenerative joint disease^12^. Lee et al*.*^13^ adopted RF to determine the rank of the risk factors related to temporomandibular disorders. ML has demonstrated the potential for predicting surgical outcomes^14^.

To our knowledge, stability prediction of 2 J-OGS surgery using ML has not been reported. Since the obvious clinical expression in patients with skeletal class III is the sagittal chin projection (Pog), the quantitative change in Pog was selected for investigation. The purpose of the present study was to identify the critical factors associated with the surgical stability of Pog by applying ML to predict relapse following 2 J-OGS.

## Methods

### Subjects

The study sample consisted of 319 adult Korean patients diagnosed with skeletal class III malocclusion who underwent combined surgical orthodontic treatment and 2 J-OGS surgery at Seoul National University Dental University Hospital or Ajou University Dental Hospital, located in Republic of Korea, between 2006 and 2017. The inclusion criteria were as follows; (1) patients who had undergone 2 J-OGS surgery, Le Fort I osteotomy in the maxilla, and bilateral sagittal split osteotomy in the mandible, (2) patients who underwent rigid fixation with a metal plate and monocortical screws for fixation of the osteotomized bony segments, (3) patients for whom photographs and lateral cephalograms were taken at the initial visit (T0), at least one month before the surgery (T1), at least one month after the surgery (T2), and at debonding (T3), and (5) patients who faculty orthodontists treated with more than 30 years of experience (SHB and YHK). The exclusion criteria were (1) patients who had cleft lip and/or palate or congenital craniofacial deformities, (2) patients who had a history of trauma in the craniofacial area, and (3) patients who had severe facial asymmetry (menton deviation > 5 mm), and (4) patients who underwent vertical genioplasty. Supplementary Table 1 describes the age, sex, and Pog posterior movement (1.59 ± 2.76 mm). Consequently, the final study sample included 227 patients (110 males and 117 females). This retrospective case–control study was reviewed and approved by the Institutional Review Board of Seoul National University Dental Hospital (IRB no. ERI20022) and Ajou University Hospital (IRB no. AJIRB-MED-MDB-19–039). All experimental protocols were approved by the two institutional committees. Seoul National University Dental Hospital and Ajou University Hospital IRB committees waived the need of patient informed consent. Previous studies have indicated that the major relapse after 2 J-OGS surgery occurred within 8 weeks^7^ to 1 year^5^. Thus, 1 year of follow-up was sufficient to examine relapse.

### Sample size calculation

Power analyses were conducted using Cohen's effective sample size^15^ with a significance level (α) of 0.05 and a power (1-β) of 0.9. Based on the mean and standard deviation (SD) values of postsurgical linear change in Pog from a previous study^7^, which were reported as 1.87 and 2.6 mm, respectively, sample size calculations were performed using R software (ver. 4.0.3, Vienna, Austria). The results indicated that a minimum of 20 individuals were required to achieve the desired statistical power for the study. According to Rajput's suggestion^16^, a suitable sample size in machine learning algorithms should have an effective size greater than 0.5 and an ML accuracy of over 80%. Additionally, Rajput indicated that increasing the sample size beyond the threshold point would not significantly improve performance. In this study, the standardized effect size was 1.14, which exceeds the threshold of 0.5, indicating a substantial effect size. Therefore, among the machine learning algorithms used in this study, those that demonstrate an accuracy of more than 80% can be considered acceptable in terms of their performance.

### Landmarks and variables used in this study

**Figure 1:**
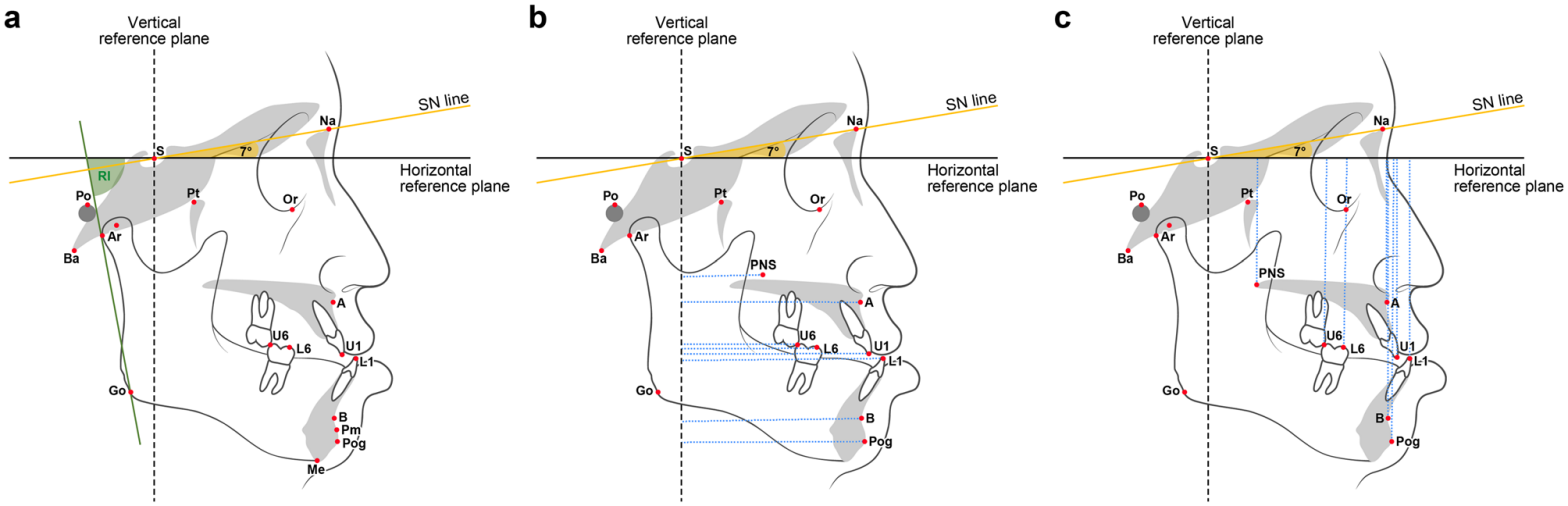
Landmarks, reference lines, and definitions of linear and angular measurements. Linear measurements: 1. A_x (mm), the horizontal distance from point A to VRP. 2. PNS_x (mm), the horizontal distance from PNS to VRP. 3. U1_x (mm), the horizontal distance from U1E to VRP. 4. U6_x (mm), the horizontal distance from U6MBC to VRP. 5. A_y (mm), the vertical distance from point A to HRP. 6. PNS_y (mm), the vertical distance from PNS to HRP. 7. U1_y(mm), the vertical distance from U1E to HRP. 8. U6_y (mm), the vertical distance from U6MBC to HRP. 9. B_x (mm), the horizontal distance from point B to VRP. 10. Pog_x (mm), the horizontal distance from Pog to VRP. 11. L1_x (mm), the horizontal distance from L1E to VRP. 12. L6_x (mm), the horizontal distance from L6MBC to VRP. 13. B_y (mm), the vertical distance from point B to HRP. 14. Pog_y(mm), the vertical distance from Pog to HRP. 15. L1_y (mm), the vertical distance from L1E to HRP. 16. L6_y (mm), the vertical distance from L6MBC to HRP.

**Figure 2:**
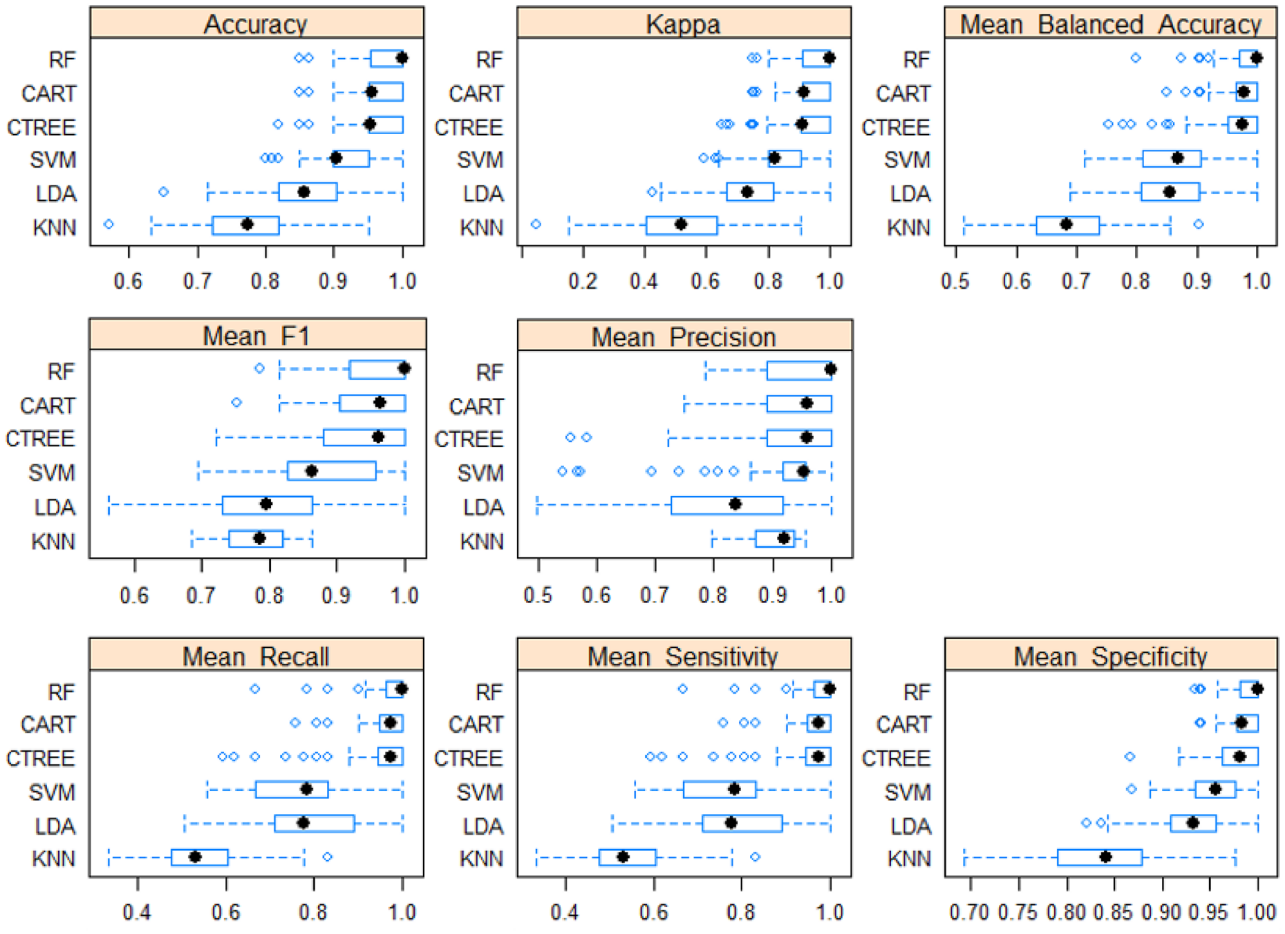
Comparison of machine learning models (Dependent: RANK—HS/S/N). Scale 1.00 indicates the best metric performance.

Figures 1 and 2 illustrated the definitions of the landmarks and linear and angular variables. Fifty-five linear and angular skeletal and dental surgical movements (T2-T1) were measured, of which 16 were calibrated relative to the horizontal and vertical reference planes for further analysis of linear changes to assess the magnitude of surgical movement. Postoperative relapse was estimated by measuring Pog movement (T3-T2). The identification of landmarks and measurement of variables were performed by a single operator (YHK).

### Intra-examiner reliability assessment

To evaluate intra-examiner reliability, the same investigator (YHK) re-evaluated all variables from 20 randomly selected subjects one month after the initial measurement. After conducting paired t-tests, no significant differences were observed between the first and second measurements. As a result, the first set of variables was used for subsequent statistical analyses.

### Statistical analyses

The normality of the data distribution for each variable was assessed using the Shapiro–Wilk test. Statistical analysis was conducted among groups using a one-way analysis of variance and the Kruskal–Wallis test. A Bonferroni post-hoc analysis was performed. Statistical analysis was performed using R version 4.2.2. A significance level of p < 0.05 was established for all statistical tests.

### ML algorithms

Six ML approaches were utilized to identify factors contributing to Pog relapse, and these algorithms were compared to determine the optimal method for prediction, classification, and regression trees (CART)^17^, conditional inference tree (CTREE)^18^, linear discriminant analysis^19^, support vector machine^20^, KNN^21^, RF^22^. A tenfold cross-validation was performed, repeating the process ten times to further reduce the variance in the results. The literature^23^ supporting k-fold cross-validation indicates that it is an effective resampling technique to mitigate overfitting in machine learning models. Cross-validation is particularly useful when dealing with limited data samples. In the k-fold cross-validation process, the dataset is partitioned into k subsets, or "folds," with equal sizes. During the evaluation phase, the model is trained and tested k times. In each iteration, one fold is held out as the test set, while the remaining (k-1) folds are used for training the model. This procedure ensures that the model is assessed on different subsets of data, which helps to provide a more robust evaluation of its performance^24,25^. The primary advantage of k-fold cross-validation is that it allows the model to be trained and tested on various data partitions, thereby reducing the risk of overfitting. Overfitting occurs when a model becomes too specialized to the training data and performs poorly on new, unseen data. By repeatedly evaluating the model on different data subsets, k-fold cross-validation helps to identify whether the model generalizes well across various data distributions. This technique provides a more reliable estimate of the model's performance metrics, such as accuracy, precision, recall, and F1 score, compared to a single train-test split evaluation. Moreover, it aids in optimizing hyperparameters and selecting the best model architecture that yields better generalization to unseen data. In summary, k-fold cross-validation is a valuable tool for machine learning model evaluation, particularly when dealing with limited data and aiming to avoid overfitting. Its implementation can lead to more robust and accurate models by ensuring better generalization across different data samples. The training and testing set consisted of 207 and 20 samples, respectively.

### Metrics

The metric evaluation included accuracy, kappa, mean balanced accuracy, mean F1 score, mean recall, mean sensitivity, and mean specificity.

### Ethics declaration

The study design followed the Declaration of Helsigki principles and was approved by SNUDH and Ajou University Hospital. his retrospective case–control study was reviewed and approved by the Institutional Review Board of Seoul National University Dental Hospital (IRB no. ERI20022) and Ajou University Hospital (IRB no. AJIRB-MED-MDB-19–039). The IRB Committee waived the requirement for obtaining patient consent from both institutions.

## Results

**Figure 3:**
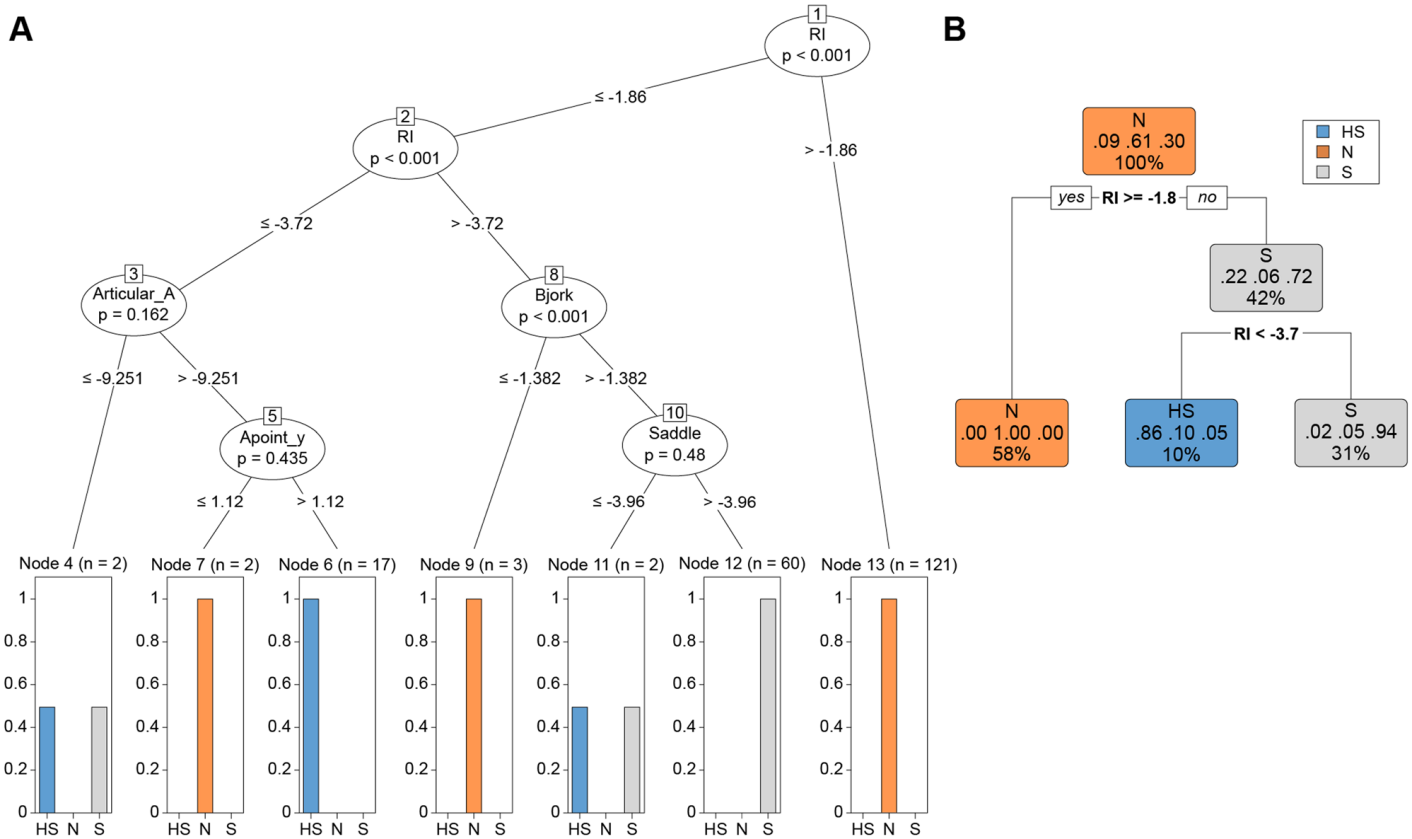
Decision Tree of Conditional Inference (CTREE). (**A**) Decision Tree of Conditional Inference (CTREE). RI, Articular angle, Bjork sum, Apoint_y (A_y), and Saddle angle were chosen for the classification. (**B**) Critical values of Classification and Regression Trees (CART) (Complexity parameter = 0.176).

Based on previous studies^2–4^, the training set was classified into three subgroups based on the rank of relapse; highly significant (HS, n = 19) relapse, which was defined as greater than 4 mm of relapse; significant (S, n = 62) relapse, which was defined as a relapse ranging between 2 and 4 mm, and insignificant (N, n = 126) relapse, which was < 2 mm. The evaluation involved calculating the position of the Pog between T3 (debonding) and T2 (surgery). The differences in cephalometric variables among the three groups in the training set (n = 207) are presented in Supplementary Table 2. Bjork sum, articular angle, gonial angle, lower anterior–posterior height ratio (ANS-Me/N-Me), FMA, SN to MP, SNA, FM_UOP, and A-point to vertical reference plane VRP displayed statistically significant differences (Fig. 1). The metrics evaluation among the ML models was shown in Fig. 2 and summarized in Table 1. A scale close to 1.0 indicated a higher prediction level. The significance of the differences between the metric distributions of the different ML algorithms was shown in Table 2. Each number indicated the difference between the algorithms, and p-values were described. For example, in accuracy, the mean difference between CART and CTREE was 0.008, obtained by subtracting them in Table 2 (|0.966–0.958|= 0.008). In general, RF presented the most significant difference. The performance metrics of the ML algorithms in the testing set (n = 20) were compared in Table 3. CART, CTREE, and RF displayed better prediction results. For example, RF predicted a sagittal chin point (Pog) surgical relapse of more than 2 mm 95% (19/20), and considering the classification between HS and S, 90% (18/20) was the same as the actual outcomes (Supplementary Table 2). In RF, "VarImp" stands for "variable importance." The variable importance measures the relative importance of each predictor variable in the RF model. The six important head variables were RI, articular angle, Bjork sum, gonial angle, Sn to MP, and FMA. (Supplementary Fig. 1). Although RF predicted the rank of relapse and found critical variables, quantitative critical points can be obtained from Decision Tree models, which also visualize the prediction process to understand the process easily (CTREE, Fig. 3a and CART, Fig. 3b). In Fig. 3a, the prediction model of CTREE was illustrated. The first step was evaluating the amount of CW rotation of the ramus to predict the Pog relapse rank, N, S, and HS. No significant relapse was forecasted if it was less than 1.86 degrees (− 1.86). When more than 1.86°of CW rotation occurred during surgery, the next step was to evaluate whether it was more or less than 3.72. The third step was determining whether the articular angle changed by more than 9.25°in the same direction. If so, the fifth step estimated the increased vertical position of point A (Apoint_y). An HS relapse was anticipated if it was more than 1.12 mm. CART (Fig. 3b) revealed that the CW rotation of the ramus with critical points of 1.8° and 3.7° was essential for forecasting the relapse rank.

**Table 1:** Performance metrics of machine learning algorithms.

		Min	1st.Qu	Median	Mean	3rd.Qu	Max	NA.s
Accuracy	CART	0.850	0.950	0.955	0.966	1.000	1.000	0
Accuracy	CTREE	0.818	0.950	0.952	0.958	1.000	1.000	0
Accuracy	LDA	0.650	0.818	0.857	0.860	0.905	1.000	0
Accuracy	SVM	0.800	0.900	0.905	0.912	0.951	1.000	0
Accuracy	KNN	0.571	0.724	0.773	0.776	0.818	0.950	0
Accuracy	RF	0.850	0.952	1.000	0.974	1.000	1.000	0
Kappa	CART	0.752	0.911	0.918	0.939	1.000	1.000	0
Kappa	CTREE	0.648	0.906	0.914	0.923	1.000	1.000	0
Kappa	LDA	0.426	0.667	0.732	0.740	0.820	1.000	0
Kappa	SVM	0.592	0.801	0.821	0.832	0.907	1.000	0
Kappa	KNN	0.050	0.405	0.521	0.521	0.634	0.906	0
Kappa	RF	0.752	0.912	1.000	0.953	1.000	1.000	0
Mean_Balanced_Accuracy	CART	0.849	0.967	0.978	0.975	1.000	1.000	0
Mean_Balanced_Accuracy	CTREE	0.755	0.951	0.977	0.964	1.000	1.000	0
Mean_Balanced_Accuracy	LDA	0.690	0.809	0.855	0.851	0.905	1.000	0
Mean_Balanced_Accuracy	SVM	0.712	0.810	0.868	0.865	0.906	1.000	0
Mean_Balanced_Accuracy	KNN	0.513	0.634	0.683	0.692	0.739	0.905	0
Mean_Balanced_Accuracy	RF	0.801	0.973	1.000	0.977	1.000	1.000	0
Mean_F1	CART	0.753	0.907	0.964	0.949	1.000	1.000	0
Mean_F1	CTREE	0.722	0.881	0.961	0.945	1.000	1.000	3
Mean_F1	LDA	0.563	0.730	0.796	0.809	0.864	1.000	20
Mean_F1	SVM	0.694	0.828	0.863	0.876	0.957	1.000	39
Mean_F1	KNN	0.686	0.741	0.785	0.783	0.820	0.863	87
Mean_F1	RF	0.786	0.919	1.000	0.956	1.000	1.000	1
Mean_Precision	CART	0.750	0.889	0.958	0.948	1.000	1.000	0
Mean_Precision	CTREE	0.556	0.889	0.958	0.939	1.000	1.000	1
Mean_Precision	LDA	0.498	0.733	0.838	0.806	0.917	1.000	12
Mean_Precision	SVM	0.542	0.917	0.952	0.912	0.956	1.000	35
Mean_Precision	KNN	0.795	0.871	0.922	0.903	0.938	0.956	87
Mean_Precision	RF	0.786	0.889	1.000	0.956	1.000	1.000	1
Mean_Recall	CART	0.760	0.951	0.974	0.964	1.000	1.000	0
Mean_Recall	CTREE	0.593	0.944	0.974	0.949	1.000	1.000	0
Mean_Recall	LDA	0.504	0.712	0.778	0.774	0.889	1.000	0
Mean_Recall	SVM	0.556	0.667	0.786	0.775	0.833	1.000	0
Mean_Recall	KNN	0.333	0.474	0.529	0.546	0.598	0.833	0
Mean_Recall	RF	0.667	0.967	1.000	0.966	1.000	1.000	0
Mean_Sensitivity	CART	0.760	0.951	0.974	0.964	1.000	1.000	0
Mean_Sensitivity	CTREE	0.593	0.944	0.974	0.949	1.000	1.000	0
Mean_Sensitivity	LDA	0.504	0.712	0.778	0.774	0.889	1.000	0
Mean_Sensitivity	SVM	0.556	0.667	0.786	0.775	0.833	1.000	0
Mean_Sensitivity	KNN	0.333	0.474	0.529	0.546	0.598	0.833	0
Mean_Sensitivity	RF	0.667	0.967	1.000	0.966	1.000	1.000	0
Mean_Specificity	CART	0.939	0.978	0.983	0.985	1.000	1.000	0
Mean_Specificity	CTREE	0.867	0.964	0.982	0.979	1.000	1.000	0
Mean_Specificity	LDA	0.821	0.909	0.933	0.928	0.956	1.000	0
Mean_Specificity	SVM	0.869	0.935	0.956	0.954	0.976	1.000	0
Mean_Specificity	KNN	0.693	0.792	0.841	0.839	0.879	0.976	0
Mean_Specificity	RF	0.935	0.981	1.000	0.989	1.000	1.000	0

Pre-processing: centered (55), scaled (55), Resampling: Cross-Validated (tenfold, repeated 10 times).

*CART* classification and regression trees (Complexity parameter = 0.176), *CTREE* conditional inference tree (mincriterion = 0.9), *LDA* linear discriminant analysis, *SVM* support vector machines (sigma = 0.01225348 and C = 2), *KNN* K-nearest neighbor (k = 13), *RF* Random Forest (mtry = 28), *By* R 4.2.2 with package 'caret', 207 samples 55 predictor 3 classes: ‘HS’, ‘S,’ ‘N’, *N* No significant relapse, *S* significant relapse, *HS* highly significant relapse.

**Table 2:** The significance of the differences between the metric distributions of different machine learning algorithms.

		CART	CTREE	LDA	SVM	KNN	RF
Accuracy	CART		0.008	0.106	0.054	0.190	− 0.008
Accuracy	CTREE	**0.004**		0.098	0.046	0.182	− 0.016
Accuracy	LDA	** < 0.001**	** < 0.001**		− 0.052	0.084	− 0.114
Accuracy	SVM	** < 0.001**	** < 0.001**	** < 0.001**		0.136	− 0.062
Accuracy	KNN	** < 0.001**	** < 0.001**	** < 0.001**	** < 0.001**		− 0.198
Accuracy	RF	**0.005**	** < 0.001**	** < 0.001**	** < 0.001**	** < 0.001**	
Kappa	CART		0.016	0.199	0.107	0.418	− 0.014
Kappa	CTREE	**0.005**		0.183	0.091	0.403	− 0.030
Kappa	LDA	** < 0.001**	** < 0.001**		− 0.092	0.220	− 0.213
Kappa	SVM	** < 0.001**	** < 0.001**	** < 0.001**		0.312	− 0.121
Kappa	KNN	** < 0.001**	** < 0.001**	** < 0.001**	** < 0.001**		− 0.433
Kappa	RF	**0.009**	** < 0.001**	** < 0.001**	** < 0.001**	** < 0.001**	
Mean_Balanced_Accuracy	CART		0.011	0.124	0.110	0.283	− 0.003
Mean_Balanced_Accuracy	CTREE	**0.023**		0.113	0.099	0.272	− 0.014
Mean_Balanced_Accuracy	LDA	** < 0.001**	** < 0.001**		− 0.013	0.159	− 0.126
Mean_Balanced_Accuracy	SVM	** < 0.001**	** < 0.001**	1.000		0.172	− 0.113
Mean_Balanced_Accuracy	KNN	** < 0.001**	** < 0.001**	** < 0.001**	** < 0.001**		− 0.285
Mean_Balanced_Accuracy	RF	1.000	** < 0.001**	** < 0.001**	** < 0.001**	** < 0.001**	
Mean_F1	CART		0.006	0.142	0.076	0.161	− 0.007
Mean_F1	CTREE	**0.031**		0.137	0.074	0.158	− 0.013
Mean_F1	LDA	** < 0.001**	** < 0.001**		− 0.069	0.028	− 0.149
Mean_F1	SVM	** < 0.001**	** < 0.001**	** < 0.001**		0.090	− 0.083
Mean_F1	KNN	**0.001**	**0.001**	1.000	0.156		− 0.170
Mean_F1	RF	** < 0.001**	** < 0.001**	** < 0.001**	** < 0.001**	**0.001**	
Mean_Precision	CART		0.009	0.143	0.037	0.037	− 0.009
Mean_Precision	CTREE	0.287		0.132	0.025	0.035	− 0.018
Mean_Precision	LDA	** < 0.001**	** < 0.001**		− 0.100	− 0.093	− 0.151
Mean_Precision	SVM	0.256	1.000	** < 0.001**		0.011	− 0.045
Mean_Precision	KNN	1.000	1.000	0.173	1.000		− 0.047
Mean_Precision	RF	** < 0.001**	**0.001**	** < 0.001**	0.058	1.000	
Mean_Recall	CART		0.016	0.190	0.189	0.419	− 0.002
Mean_Recall	CTREE	0.063		0.174	0.173	0.403	− 0.017
Mean_Recall	LDA	** < 0.001**	** < 0.001**		− 0.001	0.229	− 0.192
Mean_Recall	SVM	** < 0.001**	** < 0.001**	1.000		0.230	− 0.191
Mean_Recall	KNN	** < 0.001**	** < 0.001**	** < 0.001**	** < 0.001**		− 0.420
Mean_Recall	RF	1.000	**0.003**	** < 0.001**	** < 0.001**	** < 0.001**	
Mean_Sensitivity	CART		0.016	0.190	0.189	0.419	− 0.002
Mean_Sensitivity	CTREE	0.063		0.174	0.173	0.403	− 0.017
Mean_Sensitivity	LDA	** < 0.001**	** < 0.001**		− 0.001	0.229	− 0.192
Mean_Sensitivity	SVM	** < 0.001**	** < 0.001**	1.000		0.230	− 0.191
Mean_Sensitivity	KNN	** < 0.001**	** < 0.001**	** < 0.001**	** < 0.001**		− 0.420
Mean_Sensitivity	RF	1.000	**0.003**	** < 0.001**	** < 0.001**	** < 0.001**	
Mean_Specificity	CART		0.006	0.057	0.031	0.147	− 0.004
Mean_Specificity	CTREE	**0.005**		0.051	0.025	0.140	− 0.010
Mean_Specificity	LDA	** < 0.001**	** < 0.001**		− 0.026	0.089	− 0.061
Mean_Specificity	SVM	** < 0.001**	** < 0.001**	** < 0.001**		0.115	− 0.035
Mean_Specificity	KNN	** < 0.001**	** < 0.001**	** < 0.001**	** < 0.001**		− 0.150
Mean_Specificity	RF	**0.026**	** < 0.001**	** < 0.001**	** < 0.001**	** < 0.001**	

Significant values are in bold.

p-value adjustment: Bonferroni.

Upper diagonal: estimates of the difference.

Lower diagonal: p-value for H0: difference = 0.

**Table 3:** Performance metrics of machine learning algorithms for Testing data set (n = 20).

n = 20	Overall statistics	Statistics by class	Sensitivity	Specificity	Precision	Recall	F1	Prevalence	Detection rate	Detection prevalence	Balanced accuracy
**Test_CART**	**Accuracy : 1.000 (0.832, 1.000)**	Class: HS	1.000	1.000	1.000	1.000	1.000	0.150	0.150	0.150	1.000
**Test_CART**	**P-value [Acc > NIR]: < 0.001**	Class: N	1.000	1.000	1.000	1.000	1.000	0.550	0.550	0.550	1.000
**Test_CART**	**Kappa: 1.000**	Class: S	1.000	1.000	1.000	1.000	1.000	0.300	0.300	0.300	1.000
**Test_CTREE**	**Accuracy: 1.000 (0.832, 1.000)**	Class: HS	1.000	1.000	1.000	1.000	1.000	0.150	0.150	0.150	1.000
**Test_CTREE**	**P-value [Acc > NIR]: < 0.001**	Class: N	1.000	1.000	1.000	1.000	1.000	0.550	0.550	0.550	1.000
**Test_CTREE**	**Kappa: 1.000**	Class: S	1.000	1.000	1.000	1.000	1.000	0.300	0.300	0.300	1.000
Test_KNN	Accuracy: 0.850 (0.621, 0.968)	Class: HS	0.000	1.000	NA	0.000	NA	0.150	0.000	0.000	0.500
Test_KNN	P-value [Acc > NIR]: 0.005	Class: N	1.000	0.889	0.917	1.000	0.957	0.550	0.550	0.600	0.944
Test_KNN	Kappa: 0.727	Class: S	1.000	0.857	0.750	1.000	0.857	0.300	0.300	0.400	0.929
Test_LDA	Accuracy: 0.900 (0.683, 0.9877)	Class: HS	0.667	1.000	1.000	0.667	0.800	0.150	0.100	0.100	0.833
Test_LDA	P-value [Acc > NIR]: < 0.001	Class: N	1.000	0.889	0.917	1.000	0.957	0.550	0.550	0.600	0.944
Test_LDA	Kappa: 0.823	Class: S	0.833	0.929	0.833	0.833	0.833	0.300	0.250	0.300	0.881
**Test_RF**	**Accuracy: 1.000 (0.832, 1.000)**	Class: HS	1.000	1.000	1.000	1.000	1.000	0.150	0.150	0.150	1.000
**Test_RF**	**P-value [Acc > NIR]: < 0.001 **	Class: N	1.000	1.000	1.000	1.000	1.000	0.550	0.550	0.550	1.000
**Test_RF**	**Kappa: 1.000**	Class: S	1.000	1.000	1.000	1.000	1.000	0.300	0.300	0.300	1.000
Test_SVM	Accuracy: 0.900 (0.683, 0.9877)	Class: HS	0.667	1.000	1.000	0.667	0.800	0.150	0.100	0.100	0.833
Test_SVM	P-value [Acc > NIR]: < 0.001	Class: N	1.000	0.889	0.917	1.000	0.957	0.550	0.550	0.600	0.944
Test_SVM	Kappa: 0.823	Class: S	0.833	0.929	0.833	0.833	0.833	0.300	0.250	0.300	0.881

No information rate: 0.550.

## Discussion

This study aimed to predict the stability of sagittal chin projection (Pog) following 2 J-OGS surgery using ML. The changes in Pog during surgery between the preoperative (T1) and postoperative (T2) stages were used to predict the change in Pog at the debonding stage (T3). This study employed ML algorithms to identify the critical factors associated with the surgical stability of Pog. In agreement with earlier research^6,7^, our study emphasizes the significance of changes in Pog relapse between the pre-operative and post-operative stages as indicators of surgical instability. This supports the idea that alterations in the proximal segment of the mandible in the clockwise (CW) direction during surgery and counterclockwise (CCW) direction in the retention period are crucial factors in determining the stability of Pog. The application of ML algorithms to predict surgical stability in orthodontics and dental orthognathic surgery has been gaining interest in recent years. In this context, our study builds upon previous work by Jung^9^, Etemd^10^, and Li^11^ successfully utilized AI techniques to classify extraction versus non-extraction cases, rank factors determining extraction, and distinguish between extraction patterns, respectively. The current study expands this research with a comparable performance by employing ML algorithms to predict Pog stability following 2 J-OGS surgery, which has not been previously explored.

In this study, a tenfold cross-validation method was used to evaluate the predictive performance of the ML model. The performances of six popular ML algorithms were compared by adopting multiple evaluation metrics. Since the sample number of each group was different and the HS group had the smallest number (n = 19), the mean balance accuracy, precision, recall, and sensitivity were also investigated to account for the class imbalance. In the current study, the "false negative" detection was clinically critical since the prediction of relapse should not exclude those patients who will relapse. On the other hand, the "false positive" of the HS and S groups were not as significant as the "false negative." Therefore, the mean balanced precision, recall, and sensitivity, useful metrics when the cost of false-negative prediction is high, were utilized and examined (Fig. 2). CART and CTREE performed better than the others, and RF displayed the best scores. For example, RF exhibited the highest mean balanced accuracy, followed by CART, CTREE, Support Vector Machine, Linear Discriminant Analysis, and KNN (Table 1). Statistical differences were examined among the ML models (Table 2). For example, the mean balanced accuracy of RF differed from the others, except for CART. Table 3 demonstrates the testing set data results, which indicate that RF, CART, and CTREE also exhibit superior performance. Therefore, the results of these three algorithms were investigated further (Table 3).

As shown in Supplementary Table 2, RF was predicted correctly in 18/20 samples. Case number four underwent 4.78 mm relapse (HS in reality), but it was predicted to be in the S group, which was inaccurate but partially correct regarding whether relapse occurred. Case number five showed a 2 mm Pog backward movement, but that number was incorrect. A unique feature of RF is that it reveals an important variable (Supplementary Fig. 1); an essential variable that affected the rank prediction was RI, followed by the articular angle, Bjork sum, gonial angle, Sn to MP, and FMA. These variables were all related to the vertical increment during surgery, implicating the importance of maintaining the vertical dimension in the mandible. The composition of the decision-making triage is illustrated in Fig. 3. CTREE forecasted that the first and second critical numbers of RI CW rotation were 1.86 (S) and 3.72 (HS), respectively. The articular angle and Bjork sum were nominated in the next tree, followed by A point vertical and a saddle angle increment. The most crucial advantage of decision trees is that they suggest critical numbers. The exact numbers were acquired using CART regarding the RI CW rotation (Fig. 3b).

The present study has several limitations. The first is the overfitting of the ML algorithms. Overfitting is a common problem in ML. A model is trained to fit the training data so closely that it starts memorizing instead of generalizing and identifying patterns. When a model overfits, it performs very well on the training data; however, its performance on new, unseen data is poor. This phenomenon occurs when the model is overly tailored to the training dataset, leading to reduced generalizability and accuracy when making predictions on new, unseen data^26^. Furthermore, this study only collected samples from two universities, two oral surgeons who operated on the surgery, and two orthodontists who performed orthodontic treatment. Considering the different treatment plans, techniques, and ethnic backgrounds, other institutions may have different predictions. Nonetheless, it may be more appropriate to make predictions based on data from each institution, given that most institutions likely employ specific surgical techniques and orthodontic mechanics. The second limitation is that Pog was the only measurement. Other measurements, such as the maxillary occlusal plane^27^, vertical bony step^28^ and points A, B, etc., should be addressed in future studies.

This study provides valuable insights into ML's application of ML in predicting Pog stability after 2 J-OGS surgery. The findings of this study indicate that the ML model developed could be used to predict the relapse of Pog accurately, suggesting the critical number of variables associated with the surgical stability of Pog. The clinical implication of the current study was that ML applications could be used to identify patients at high risk of surgical relapse and develop appropriate postoperative management strategies to improve surgical stability. The model's accuracy in predicting Pog's relapse could reduce the need for further surgical procedures, reducing the treatment cost and duration.

## Conclusions

The primary objective of this study was to utilize ML algorithms to predict sagittal chin projection (Pog) stability after 2 J-OGS surgery and identify the key factors contributing to surgical stability. Changes in Pog relapse with mandibular CW rotation during surgery served as indicators of surgical instability. RF, CART, and CTREE demonstrated the most robust predictive performances of the six ML algorithms assessed in this study. The study revealed that a CW rotation of more than 3.7 and 1.8 degrees of RI CW rotation was the most significant risk factor for HS (≥ 4) and S (≥ 2 and < 4) Pog relapse, respectively. The findings of this study suggest that ML algorithms, mainly RF and decision-tree models, are practical tools for predicting surgical stability. Additionally, decision tree models enable the visualization of the prediction process using a triage illustration.

### Supplementary Information

Supplementary Figure 1.

Supplementary Legends.

Supplementary Table 1.

Supplementary Table 2.

## Data Availability

The test set data can be obtained via github (https://github.com/pfChae/The-prediction-of-sagittal-chin-point-relapse-following-double-jaw-surgery-using-machine-learning).
