# Author Correction: The prediction of sagittal chin point relapse following two-jaw surgery using machine learning

**DOI:** 10.1038/s41598-024-53035-x

**Published:** 2024-02-02

**Authors:** Young Ho Kim, Inhwan Kim, Yoon-Ji Kim, Minji Kim, Jin-Hyoung Cho, Mihee Hong, Kyung-Hwa Kang, Sung-Hoon Lim, Su-Jung Kim, Namkug Kim, Jeong Won Shin, Sang-Jin Sung, Seung-Hak Baek, Hwa Sung Chae

**Affiliations:** 1 Department of Orthodontics, Institute of Oral Health Science, Ajou University School of Medicine, Suwon, South Korea; 2 Department of Convergence Medicine, Asan Medical Center, Asan Medical Institute of Convergence Science and Technology, University of Ulsan College of Medicine, Seoul, Korea; 3 Department of Orthodontics, Asan Medical Center, University of Ulsan College of Medicine, Seoul, Korea; 4 Department of Orthodontics, College of Medicine, Ewha Woman’s University, Seoul, Korea; 5 Department of Orthodontics, Chonnam National University School of Dentistry, Gwangju, Korea; 6 Department of Orthodontics, School of Dentistry, Kyungpook National University, Daegu, Korea; 7 Department of Orthodontics, School of Dentistry, Wonkwang University, Iksan, Korea; 8 Department of Orthodontics, College of Dentistry, Chosun University, Gwangju, Korea; 9 Department of Orthodontics, Kyung Hee University School of Dentistry, Seoul, Korea; 10 Department of Orthodontics, School of Dentistry, Dental Research Institute, Seoul National University, Seoul, South Korea; 11 Department of Orthodontics, Gwangmyeong Hospital, Chungang University, Gwangmyeong, Korea

Correction to: *Scientific Reports* 10.1038/s41598-023-44207-2, published online 09 October 2023

The original version of this Article contained an error in the spelling of the author Minji Kim, which was incorrectly given as Minji Ki.

The original Article has been corrected.

