# Supplementary figures and images for: The prediction of sagittal chin point relapse following two-jaw surgery using machine learning
Source: Sci Rep. 2023 Oct 9;13:17005. doi: 10.1038/s41598-023-44207-2 (PMC10562368; doi:10.1038/s41598-023-44207-2)

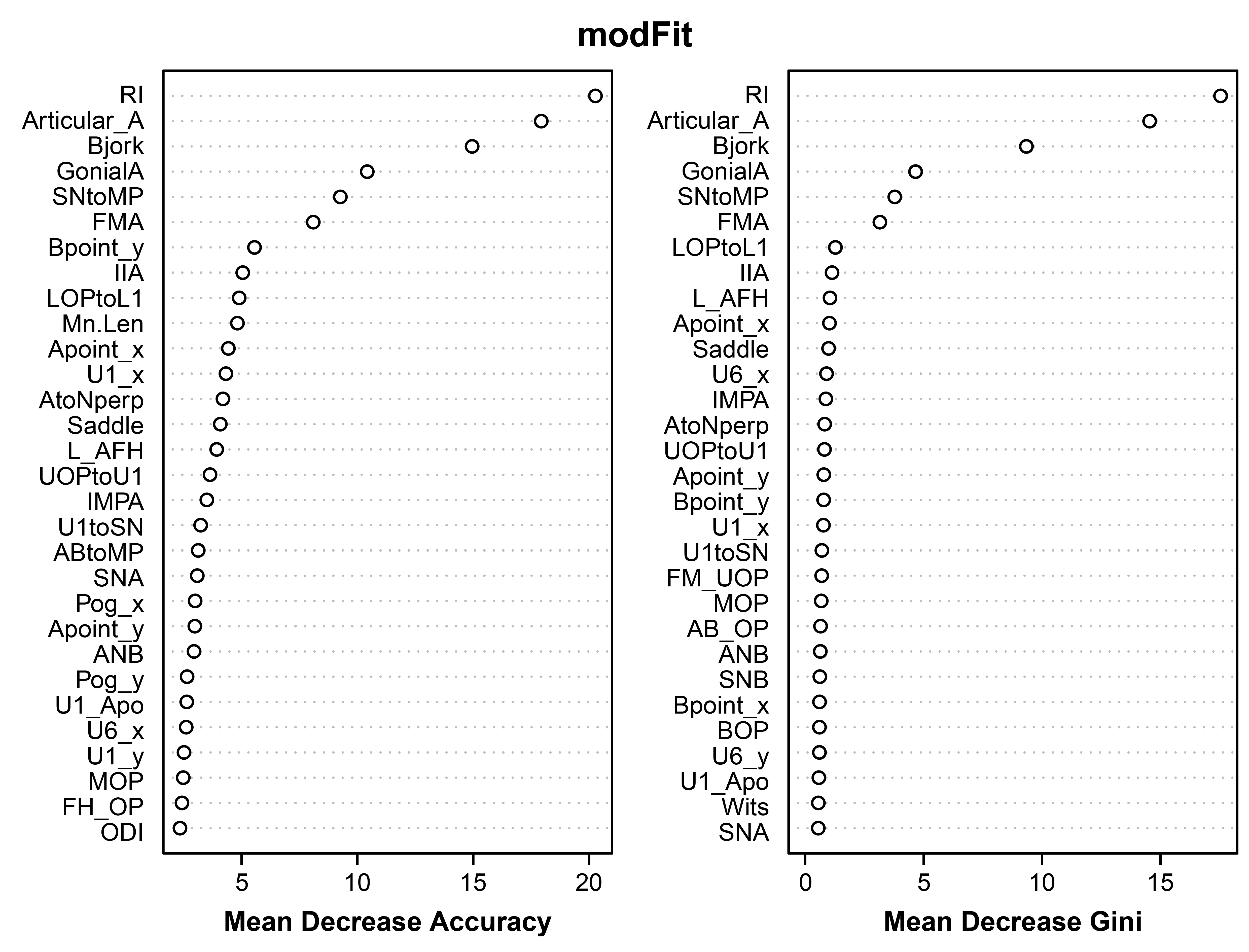

Supplement: Supplementary file 1 — Supplementary Figure 1. [file 41598_2023_44207_MOESM1_ESM.tif]
