# Supplementary material for: The prediction of sagittal chin point relapse following two-jaw surgery using machine learning
Source: Sci Rep. 2023 Oct 9;13:17005. doi: 10.1038/s41598-023-44207-2 (PMC10562368; doi:10.1038/s41598-023-44207-2)
Supplement: Supplementary file 2 — Supplementary Legends. [file 41598_2023_44207_MOESM2_ESM.docx]

**Supplementary Figure 1. Var important variables resulted from the random forest.**

Higher scores indicate important variables.
