# Supplementary material for: The prediction of sagittal chin point relapse following two-jaw surgery using machine learning
Source: Sci Rep. 2023 Oct 9;13:17005. doi: 10.1038/s41598-023-44207-2 (PMC10562368; doi:10.1038/s41598-023-44207-2)
Supplement: Supplementary file 3 — Supplementary Table 1. [file 41598_2023_44207_MOESM3_ESM.docx]

**Supplementary Table 1. Demographic data**

| Age | T0 | 21.1±3.6 |
| --- | --- | --- |
| (Years) | T1 | 22.3±3.2 |
|  | T2 | 22.5±3.3 |
|  | T3 | 23.6±3.85 |
| Gender | Male | 110 |
|  | Female | 117 |
| Pog relapse (mm) |  | 1.59±1.76 |

T0, initial visit; T1, at least 1 month before two-jaw surgery; T2, at least 1 month after two-jaw surgery; T3, debonding
