# Supplementary material for: The prediction of sagittal chin point relapse following two-jaw surgery using machine learning
Source: Sci Rep. 2023 Oct 9;13:17005. doi: 10.1038/s41598-023-44207-2 (PMC10562368; doi:10.1038/s41598-023-44207-2)
Supplement: Supplementary file 4 — Supplementary Table 2. [file 41598_2023_44207_MOESM4_ESM.docx]

**Supplementary Table 2. Differences in cephalometric variables between the N, S, and HS groups in the training set (N = 207)**

|  | Total (n=207) | HS (n=19) | S (n=62) | N (n=126) | p-value | A or K |
| --- | --- | --- | --- | --- | --- | --- |
| Bjork | 1.68±1.52 | 3.14±2.78 | 2.74±0.94 | 0.94±0.91 | **<0.001** | K*** |
| Saddle | 0.00±1.66 | -0.46±2.07 | 0.13±1.75 | 0.01±1.54 | 0.658 | K |
| Articular_A | -2.13±1.79 | -5.02±1.57 | -3.38±1.29 | -1.08±0.98 | **<0.001** | K*** |
| Gonial_A | 2.95±1.67 | 4.89±3.20 | 3.81±1.11 | 2.23±1.02 | **<0.001** | K*** |
| AP_FHL | -1.02±3.69 | -0.86±2.31 | -1.23±3.84 | -0.95±3.80 | 0.998 | K |
| L_AFH | -0.51±1.43 | -0.52±1.11 | -0.96±1.78 | -0.29±1.22 | **0.011** | A* |
| PPA | 2.39±3.14 | 2.30±2.75 | 2.84±3.50 | 2.18±3.00 | 0.552 | K |
| ABtoMP | 8.78±5.98 | 7.73±8.74 | 8.94±5.39 | 8.86±5.79 | 0.365 | K |
| ODI | 11.17±7.17 | 10.03±10.20 | 11.78±6.83 | 11.04±6.83 | 0.615 | A |
| FMA | 2.66±1.81 | 4.61±3.34 | 3.45±1.37 | 1.97±1.22 | **<0.001** | K*** |
| SNtoMP | 3.16±1.82 | 5.11±3.36 | 3.98±1.34 | 2.46±1.25 | **<0.001** | A*** |
| AtoNperp | 1.83±2.58 | 1.67±2.70 | 1.31±2.56 | 2.11±2.56 | 0.088 | K |
| PogtoNperp | -7.67±5.63 | -5.96±6.32 | -8.58±5.40 | -7.48±5.60 | 0.154 | K |
| SNA | 1.24±1.96 | 0.87±1.76 | 0.72±2.03 | 1.55±1.91 | **0.016** | A* |
| SNB | -3.74±2.48 | -3.58±4.35 | -4.18±2.09 | -3.54±2.27 | 0.056 | K |
| ANB | 4.98±2.81 | 4.45±5.20 | 4.90±2.50 | 5.09±2.46 | 0.201 | K |
| APDI | -9.04±6.40 | -7.64±10.15 | -8.73±5.89 | -9.41±5.94 | 0.217 | K |
| CF | 2.13±6.87 | 2.40±5.58 | 3.05±8.06 | 1.63±6.40 | 0.406 | A |
| Wits | 6.27±3.82 | 5.97±6.37 | 6.58±3.51 | 6.17±3.49 | 0.737 | A |
| F.Convexity | 10.43±6.42 | 8.88±11.04 | 10.29±5.93 | 10.72±5.72 | 0.499 | A |
| R.Ht | -3.15±4.98 | -3.21±3.38 | -3.66±4.42 | -2.89±5.44 | 0.642 | K |
| Mn.Len | -7.41±5.77 | -6.88±5.15 | -6.74±4.88 | -7.82±6.25 | 0.441 | A |
| BtoACB | -0.10±0.08 | -0.10±0.08 | -0.10±0.07 | -0.11±0.09 | 0.668 | A |
| UOPtoU1 | -0.11±4.02 | -0.26±5.75 | 0.24±3.89 | -0.25±3.79 | 0.520 | K |
| LOPtoL1 | 1.33±3.82 | 2.12±5.88 | 1.08±3.58 | 1.33±3.56 | 0.884 | K |
| U1toSN | -3.98±4.62 | -2.50±6.70 | -4.44±4.52 | -3.98±4.28 | 0.280 | A |
| U1FH | -3.68±4.71 | -1.83±5.46 | -4.05±4.45 | -3.78±4.68 | 0.187 | A |
| U1_Apo | 2.37±2.09 | 2.05±2.17 | 2.48±2.13 | 2.37±2.07 | 0.728 | K |
| U1_Stm | -1.03±1.57 | -1.08±1.51 | -1.15±1.44 | -0.97±1.65 | 0.759 | A |
| IMPA | -0.93±4.83 | -2.42±4.89 | -1.28±4.50 | -0.53±4.96 | 0.151 | K |
| L1_Apo | -4.06±2.50 | -4.20±3.32 | -4.13±2.14 | -4.01±2.54 | 0.935 | K |
| IIA | 1.96±5.98 | 2.03±6.54 | 2.70±4.95 | 1.58±6.35 | 0.252 | K |
| FM_UOP | 3.79±3.12 | 2.09±3.16 | 3.81±3.19 | 4.03±3.02 | **0.046** | K* |
| FH_OP | 3.22±2.91 | 1.79±2.89 | 3.03±2.73 | 3.52±2.96 | 0.132 | K |
| SN_OP | 3.47±3.13 | 2.61±3.73 | 3.36±3.12 | 3.65±3.03 | 0.468 | K |
| AB_OP | -7.64±6.27 | -7.85±11.30 | -7.77±5.54 | -7.55±5.60 | 0.686 | K |
| Apoint_x | 1.61±2.16 | 1.14±2.23 | 1.21±2.19 | 1.88±2.11 | **0.017** | K* |
| Apoint_y | -0.81±1.94 | -1.47±1.84 | -0.91±2.15 | -0.66±1.83 | 0.245 | K |
| PNS_x | 1.50±2.55 | 1.30±2.66 | 1.40±2.56 | 1.58±2.55 | 0.286 | K |
| PNS_y | -3.57±2.48 | -3.21±2.99 | -3.82±2.47 | -3.51±2.42 | 0.584 | K |
| Bpoint_x | -6.81±4.52 | -6.27±7.31 | -7.50±3.85 | -6.55±4.29 | 0.346 | A |
| Bpoint_y | -2.53±2.96 | -2.09±3.79 | -2.56±3.07 | -2.58±2.77 | 0.884 | K |
| Pog_x | -7.91±5.45 | -6.86±8.41 | -8.75±4.80 | -7.65±5.19 | 0.293 | A |
| Pog_y | -2.91±3.56 | -2.46±4.38 | -2.90±3.77 | -2.99±3.35 | 0.959 | K |
| U1_x | 0.16±2.51 | 0.07±2.85 | -0.30±2.88 | 0.40±2.24 | 0.157 | K |
| U1_y | -0.15±1.72 | -0.56±1.54 | -0.03±1.97 | -0.14±1.61 | 0.382 | K |
| U6_x | 1.11±2.63 | 0.91±3.12 | 0.61±2.83 | 1.38±2.42 | 0.102 | K |
| U6_y | -2.21±1.86 | -1.84±2.20 | -2.18±1.78 | -2.27±1.85 | 0.416 | K |
| L1_x | -5.98±3.81 | -5.87±5.61 | -6.67±3.29 | -5.66±3.71 | 0.187 | K |
| L1_y | -2.23±2.74 | -2.27±3.25 | -2.30±2.86 | -2.19±2.62 | 0.661 | K |
| L6_x | -5.74±3.63 | -5.36±5.78 | -5.97±3.16 | -5.68±3.46 | 0.785 | A |
| L6_y | -3.74±2.33 | -3.28±2.24 | -3.59±2.65 | -3.89±2.18 | 0.597 | K |
| MOP | 4.15±3.31 | 2.69±3.51 | 4.24±3.77 | 4.32±3.00 | 0.258 | K |
| BOP | 3.78±3.25 | 2.59±3.17 | 3.63±3.47 | 4.03±3.12 | 0.250 | K |
| RI | -1.63±1.64 | -4.58±1.59 | -2.81±0.58 | -0.59±0.90 | **<0.001** | K*** |
| Relapse | 1.59±1.76 | 4.82±1.05 | 2.96±0.57 | 0.44±0.97 | **<0.001** | A*** |

N : No significant relapse;, S : Significant relapse;, HS : Highly Significant relapse.,

A or K: Oone-way aAnalysis of variance or Kruskal-Wallis test was used to compare variables, depending on the normality.

* P<0.05, ** P<0.01, ***P<0.001
